# High-Sensitive TRBC1-Based Flow Cytometric Assessment of T-Cell Clonality in Tαβ-Large Granular Lymphocytic Leukemia

**DOI:** 10.3390/cancers14020408

**Published:** 2022-01-14

**Authors:** Noemí Muñoz-García, F. Javier Morán-Plata, Neus Villamor, Margarida Lima, Susana Barrena, Sheila Mateos, Carolina Caldas, Jacques J. M. van Dongen, Alberto Orfao, Julia Almeida

**Affiliations:** 1 Translational and Clinical Research Program, Centro de Investigación del Cáncer and IBMCC (CSIC—University of Salamanca), Cytometry Service, NUCLEUS, Department of Medicine, University of Salamanca (USAL) and Institute of Biomedical Research of Salamanca (IBSAL), 37007 Salamanca, Spain; noemimg@usal.es (N.M.-G.); fjmoranp@usal.es (F.J.M.-P.); subadelfa@usal.es (S.B.); sheilamateos@usal.es (S.M.); carolina.caldas@usal.es (C.C.); J.J.M.van_Dongen@lumc.nl (J.J.M.v.D.); orfao@usal.es (A.O.); 2 Biomedical Research Networking Centre Consortium of Oncology (CIBERONC), Instituto de Salud Carlos III, 28029 Madrid, Spain; villamor@clinic.cat; 3 Department of Pathology, Hematopathology Unit, Hospital Clínic, IDIBAPS, 08036 Barcelona, Spain; 4 Department of Hematology, Laboratory of Cytometry, Hospital de Santo António, Centro Hospitalar do Porto, 4099-001 Porto, Portugal; 5 Unit for Multidisciplinary Research in Biomedicine (UMIB), Abel Salazar Institute of Biomedical Sciences (ICBAS), University of Porto, 4050-313 Porto, Portugal; 6 Department of Immunology, Leiden University Medical Center (LUMC), 2333 Leiden, The Netherlands

**Keywords:** TRBC1, JOVI-1, TCRVβ repertoire, large granular lymphocytic leukemia, LGLL, large granular lymphocytes, LGL, Tαβ-cell maturation stages, Tαβ effector cells, flow cytometry T-cell clonality assessment

## Abstract

**Simple Summary:**

TRBC1 expression analysis by flow cytometry (FCM) has been recently proved to be a useful, simple and fast approach to assessing Tαβ-cell clonality. The aim of this study was to validate the utility of this assay specifically for the diagnosis of T-cell clonality of T-large granular lymphocytic leukemias (T-LGLL), as more mature polyclonal Tαβ large granular lymphocytes (Tαβ-LGL) show broader TRBC1^+^/TRBC1^−^ ratios vs. total Tαβ cells. Our results showed that a TRBC1-FCM assay is also a fast and easy method for detecting T-cell clonality in T-LGLL based on altered (increased or decreased) percentages of TRBC1^+^ Tαβ cells of LGL expansions (i.e., with lymphocytosis) suspected of T-LGLL, whereas in the absence of lymphocytosis (or in TαβCD4-LGLL), the detection of increased absolute cell-counts of more precisely defined subpopulations of T-LGL expressing individual TCRVβ families is required.

**Abstract:**

Flow cytometric (FCM) analysis of the constant region 1 of the T-cell receptor β chain (TRBC1) expression for assessing Tαβ-cell clonality has been recently validated. However, its utility for the diagnosis of clonality of T-large granular lymphocytic leukemia (T-LGLL) needs to be confirmed, since more mature Tαβ cells (i.e., T-LGL normal-counterpart) show broader TRBC1^+^/TRBC1^−^ ratios vs. total Tαβ cells. We compared the distribution and absolute counts of TRBC1^+^ and TRBC1^−^ Tαβ-LGL in blood containing polyclonal (*n* = 25) vs. clonal (*n* = 29) LGL. Overall, polyclonal TRBC1^+^ or TRBC1^−^ Tαβ-LGL ranged between 0.36 and 571 cells/μL (3.2–91% TRBC1^+^ cells), whereas the clonal LGL cases showed between 51 and 11,678 cells/μL (<0.9% or >96% TRBC1^+^ cells). Among the distinct TCRVβ families, the CD28^−^ effector-memory and terminal-effector polyclonal Tαβ cells ranged between 0 and 25 TRBC1^+^ or TRBC1^−^ cells/μL and between 0 and 100% TRBC1^+^ cells, while clonal LGL ranged between 32 and 5515 TRBC1^+^ or TRBC1^−^ cells/μL, representing <1.6% or >98% TRBC1^+^ cells. Our data support the utility of the TRBC1-FCM assay for detecting T-cell clonality in expansions of Tαβ-LGL suspected of T-LGLL based on altered percentages of TRBC1^+^ Tαβ cells. However, in the absence of lymphocytosis or in the case of TαβCD4-LGL expansion, the detection of increased absolute cell counts by the TRBC1-FCM assay for more accurately defined subpopulations of Tαβ-LGL-expressing individual TCRVβ families, allows the detection of T-cell clonality, even in the absence of phenotypic aberrations.

## 1. Introduction

T-cell large granular lymphocytic leukemia (T-LGLL) is a rare chronic lymphoproliferative disorder (CLPD) characterized by the clonal expansion of mature cytotoxic T cell—i.e., large granular T lymphocytes (T-LGL)—in blood and potentially also in other tissues [1,2,3]. A threshold of 2 × 10^9^ T-LGL/L of blood was initially mandatory for the diagnosis of T-LGLL, but lower LGL counts of ≥0.5–2 × 10^9^ cells/L are currently also recognized as a diagnostic criterion for T-LGLL when associated with typical clinical manifestations of the disease (i.e., cytopenias or autoimmune disorders) [4,5,6,7]. Altogether, this indicates that no single T-LGL count cut-off allows for clear-cut discrimination between neoplastic and reactive expansions of T-LGL. In turn, T-LGLL cells frequently display morphological and phenotypical features that largely overlap with their normal counterpart of (activated) terminal effector (TE), and to a lesser extent, also CD28^−^ effector memory (EM) T cells [5,8]. Further, more genetic surrogate markers of a clonal disorder, such as *STAT3* and *STAT5B* mutations, have been reported in only a fraction of between 21% and 73% of all T-LGLL patients [8,9,10]. Considering all the above, the demonstration of the clonal nature of the expanded T-LGL in the diagnostic work-up of T-LGLL still remains a challenge, particularly in cases presenting without lymphocytosis [8,11].

The evaluation of the pattern of expression of the constant region 1 of the T-cell receptor β chain (TRBC1) by flow cytometry (FCM) has been recently demonstrated to be a fast, simple, specific and accurate method for assessment of the clonal nature of Tαβ cells in patients suspected of having T-CLPD [12,13,14,15]. Thus, the monoclonal expansions of Tαβ cells typically show a monotypic/unimodal TRBC1 expression profile (either TRBC1^+^ or TRBC1^−^), whereas normal/reactive Tαβ cells consist of a (bimodal) admixture of both TRBC1^+^ and TRBC1^−^ cells [12,13,16], where TRBC1^+^ cells represent nearly 40% ± 6.7% (mean ± 1SD) of all Tαβ cells [13]. However, recent data also indicate that the percentage of TRBC1^+^ Tαβ cells may vary substantially among the different maturation-associated compartments of T cells. Thus, more mature populations of EM (particularly within the CD28^−^ subset) early-effector (EE) and TE-T cells display significantly broader ranges of TRBC1^+^ and TRBC1^−^ cells, compared to both total Tαβ cells and their naïve and central/transitional memory compartments [13]. Of note, clonal cells from T-LGLL patients, as well as most small T-LGL clones detected in otherwise healthy donors (HDc) [14,15], frequently display phenotypic profiles that match those of EM/TE cells [5,8]. Despite this, the recent evidence showing that broader percentages of TRBC1^+^ and TRBC1^−^ cells exist among more mature polyclonal Tαβ-LGL cells has not been taken into account in applying the TRBC1-FCM approach for the identification of clonal LGL cells. Here, we validated the utility of the TRBC1-FCM assay for the assessment of Tαβ-cell clonality in T-LGLL via direct comparisons with the relative distribution and absolute counts of TRBC1^+^ and TRBC1^−^ Tαβ cells among normal Tαβ-LGL.

## 2. Materials and Methods

### 2.1. Patients, Controls and Samples

A total of 54 EDTA-anticoagulated whole peripheral blood (PB) samples (from an identical number of subjects) were studied. Forty-two of them corresponded to samples included in a previously reported study [13], while the remaining 12 samples were prospectively collected between July and September 2021. From all PB samples, 17 corresponded to adult HD, 5 to HDc (identified for the first time in this study by the TRBC1-based FCM assay), 8 were collected from subjects with reactive Tαβ lymphocytosis and 24 corresponded to patients with Tαβ-LGLL (18 TαβCD8-LGLL, 5 TαβCD4-LGLL and 1 Tαβ double-negative LGLL). Additionally, residual polyclonal Tαβ cells from HDc and LGLL cases in which these could be clearly distinguished from the clonal Tαβ cells were also analyzed as reactive Tαβ cells. The mean age (±1SD) of subjects at sample collection was of 38 ± 8 years (y), 72 ± 14 y, 55 ± 19 y and 66 ± 17 y for HD, individuals with reactive Tαβ-LGL, HDc and patients with Tαβ-LGLL, respectively.

Prior to the study, all patients and controls gave their written informed consent to participate, and the study was conducted according to the guidelines of the Declaration of Helsinki after it had been approved by the local institutional Ethics Committee (University Hospital of Salamanca/IBSAL, CEIm reference number PI 2020 12 643).

### 2.2. Immunophenotypic Stainings

All samples were immunophenotyped using a direct immunofluorescence stain-and-then-lyse technique based on the EuroFlow standard operating procedures (SOP) [17,18,19]. The samples included in the previous publication [13] were stained with the anti-TRBC1 antibody (clone JOVI-1) in combination with monoclonal antibodies (Mab) recognizing maturation markers (e.g., CD27, CD28, CD45RA and CD62L; Table S1 Panel I), strictly following the EuroFlow SOP. The other samples were stained with a total of 40 different Mab reagents, including: (i) a common backbone of 12 antibodies against TRBC1, maturation-associated molecules (CD27, CD28, CD45RA and CD62L), markers which are frequently aberrantly expressed by clonal LGL (i.e., CD2, CD5 and CD7), and a set of four markers for identification of T cells and their major subsets (CD3, CD4, CD8 and TCRγδ); (ii) 24 TCRVβ Mab reagents (IOTest^®^ Beta Mark TCRVβ Repertoire Kit—Beckman-Coulter, Brea, CA, USA); and (iii) CD45 conjugated with four different fluorochromes (Table S1 Panel II). A detailed description of the staining protocol (adapted from the EuroFlow reference) [17,18,19] aimed at combining all these reagents into only two antibody combinations, ready to be measured in the flow cytometer, is provided in the Supplementary Materials, together with the precise composition of the antibody reagent panel (Protocol S1 and Table S1 Panel II). The sources and specificities of all Mab reagents used for the immunophenotypic assays described above are detailed in Table S2.

Immediately after the completion of sample preparation, stained cells were measured in a 5-laser Cytek Aurora flow cytometer (Cytek Biosciences, Fremont, CA, USA) using SpectroFlo software (version 2.1.0, Cytek Biosciences, Fremont, CA, USA). Instrument setup, calibration and daily quality control, as well as monitoring, were performed according to well-established EuroFlow protocols [18,19]. For data analysis, Infinicyt™ software (version 2.0, Cytognos, Salamanca, Spain) was used. A minimum of 500,000 target cells was acquired per sample, and ≥10 clustered cellular events were required for the definition of a cell population.

### 2.3. Calculation of the Number of TRBC1^+^ and TRBC1^−^ Cells within Different Maturation-Associated Compartments of Tαβ Cells and Their Major TCD8 and TCD4 Subsets

The percentage and absolute number of TRBC1^+^ and TRBC1^−^ cells within each of the different maturation-associated (i.e., naïve, central memory, transitional memory, CD28^+^ and CD28^−^ EM, EE and TE) populations of Tαβ cells and their major TCD8 and TCD4 subsets was investigated as previously described [13].

In addition, the percentage and absolute count of TRBC1^+^ and TRBC1^−^ Tαβ cells within each maturation stage was also calculated for Tαβ cells expressing each individual TCRVβ family.

### 2.4. Assessment of T-Cell Clonality on FACS-Sorted Cell Populations

The clonal nature of T-cell populations was confirmed in FACS-sorted cells highly purified (≥95% purity) by 3–5 mL of whole blood using a BD FACSAria-III flow cytometer (Becton Dickinson Biosciences, San Jose, CA, USA) [20,21,22] based on the presence of a single or a few dominant TRB and/or TRG gene rearrangements for the clonal cell subsets. In 2 T-LGLL patients, the T-cell clonality was further established on the purified cells through the confirmation of the presence of a *STAT3* mutation [2,9].

## 3. Results and Discussion

**Figure 1:**
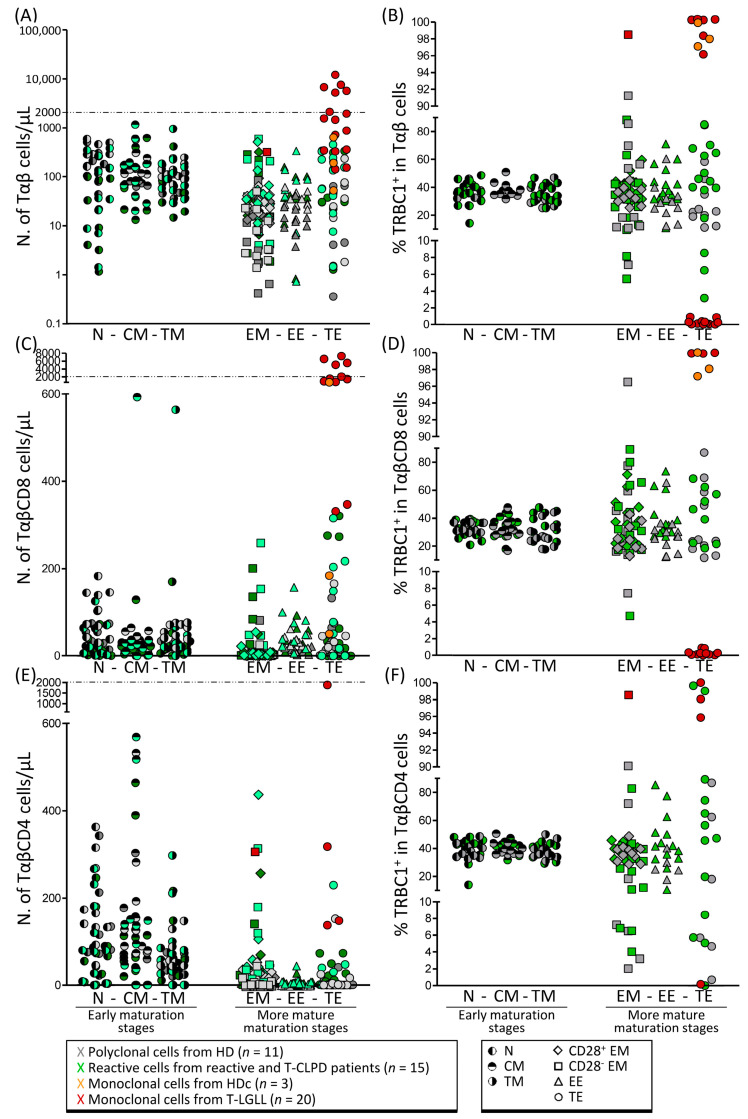
Absolute counts and relative distribution of TRBC1^+^ and TRBC1^−^ cells among normal/reactive (polyclonal) Tαβ-cell populations from normal/reactive blood classified according to their maturation stage vs. clonal Tαβ-LGL. Absolute (**A**,**C**,**E**) and relative (**B**,**D**,**F**) number of TRBC1^+^ (dark color) and TRBC1^−^ (light color) cells identified among normal (gray dots) and reactive (green dots) populations of (polyclonal) total Tαβ cells (**A**,**B**), TαβCD8 cells (**C**,**D**) and TαβCD4 cells (**E**,**F**), classified according to their maturation stage, compared to clonal Tαβ-LGL from HDc (orange dots) and T-LGLL (brown dots). Abbreviations (alphabetical order): CM, central memory; EE, early effector; EM, effector memory; HD, healthy donor; HDc, healthy donor with a Tαβ clone; LGL, large granular lymphocyte; N, naïve; N., number; PB, peripheral blood; T-CLPD, chronic lymphoproliferative disorder of T cells; T-LGLL, T-cell large granular lymphocytic leukemia; TE, terminal effector; TM, transitional memory.

In the first step, we investigated the TRBC1 expression profile of the aberrant/pathological cells from T-LGLL (*n* = 20) and HDc (*n* = 3) with that of the polyclonal EM-EE-TE T cells from HD (*n* = 11) and patients with reactive conditions (*n* = 8) in a total of 42 blood samples. Overall, the absolute count of the polyclonal TRBC1^+^ and TRBC1^−^ EM + EE + TE Tαβ cells ranged between 0.36 and 225 cells/μL in HD and between 0.71 and 571 cells/μL in reactive cases. In turn, clonal Tαβ-LGL, as identified by the TRBC1-FCM assay, ranged between 51 and 610 cells/μL in HDc and between 137 and 11,678 cells/μL in T-LGLL (Figure 1A). As expected, the absolute number of Tαβ-LGL from most T-LGLL (11/17, 65%) were significantly (*p* < 0.0001) higher than those of EM + EE + TE cells from HD and reactive cases, allowing for a clear-cut diagnosis of T-LGLL. However, while this was true for 10/11 (91%) TαβCD8-LGLL patients, in only 1/3 (33%) of TαβCD8-HDc cases and 1/5 (20%) of TαβCD4-LGLL patients, the absolute blood count of either TRBC1^+^ or TRBC1^−^ clonal T-LGL outnumbered the counts observed among their normal/reactive (mature EM + EE + TE) cell counterpart in normal/reactive blood (Figure 1A,C,E). Based on these findings, it may be concluded that the absolute count of pathological T-LGL cells identified by the TRBC1 expression profile within the whole population of Tαβ cells cannot be used as a universal (single) criterion for clear-cut detection of clonal Tαβ-LGL, particularly in T-LGLL cases that present without lymphocytosis and/or that carry relatively small (<2000 cells/μL) TαβCD4-LGL or TαβCD8-LGL clones in the blood [4,23]. In contrast, once the percentage of TRBC1^+^ cells among all aberrant/pathological vs. normal/reactive EM and TE Tαβ cells was considered, cases carrying clonal cells could be unequivocally identified and distinguished from those that only had normal polyclonal cells for all T-LGLL and HDc subjects analyzed (Figure 1B). Thus, the percentage of TRBC1^+^ cells among the more mature compartments of EM and TE Tαβ cells ranged between 7.2% and 91% in HD and between 3.2% and 89% in reactive cases, while in all HDc and T-LGLL cases, clonal cells accounted for a more extreme percentage of TRBC1^+^ cells (either <0.9% or >96%) within the corresponding maturation-associated compartment of T cells containing the expanded/suspicious (pathological) cell fraction (Figure 1B). Further subclassification of T cells into their major TαβCD8 and TαβCD4 subsets confirmed the presence of clonal cells in all TαβCD8 LGLL and HDc cases based on the percentage of TRBC1^+^ cells within their corresponding maturation-associated T-cell subset, once compared with their distribution in normal/reactive blood: all TαβCD8-LGLL showed either lower (<1.3%) or higher (>97.2%) percentages of TRBC1^+^ TE cells than normal HD (7.4–96%) and reactive (4.7–89%) EM + EE + TE cells (Figure 1D). In contrast, the fraction of TRBC1^+^ cells from all TαβCD4-LGLL (either <0.47% or >92% of suspicious cells) still overlapped with that of polyclonal TαβCD4 CD28^−^ EM or TE T cells in 4/5 (80%) reactive cases investigated (Figure 1F).

**Figure 2:**
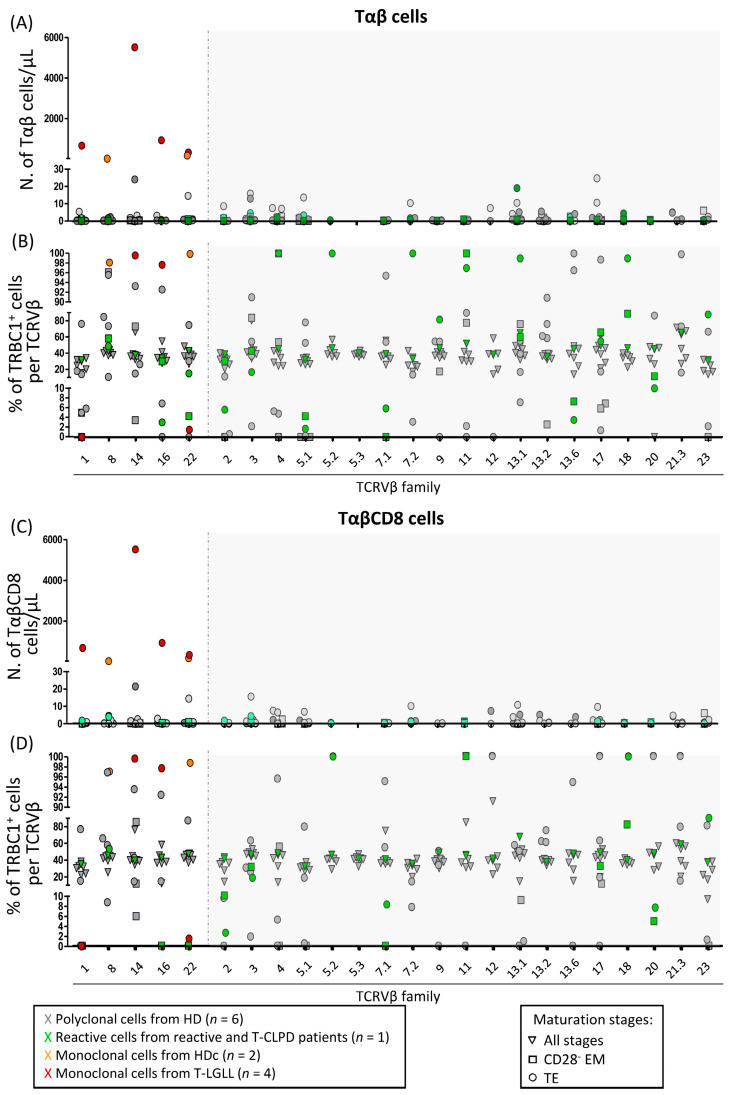
Absolute counts and relative distribution of TRBC1^+^ cells per TCRVβ family, among the more mature subsets (CD28^−^ EM and TE) of normal (*n* = 6) and reactive (*n* = 1) polyclonal Tαβ-cells vs. clonal Tαβ-LGL (*n* = 2 HDc and *n* = 4 LGLL). Absolute number (**A**,**C**) and percentage (**B**,**D**) of TRBC1^+^ cells within each TCRVβ family among total Tαβ cells (**A**,**B**) and their major TαβCD8 subset (**C**,**D**). Abbreviations (alphabetical order): EM, effector memory; HD, healthy donor; HDc, healthy donor with a T-LGL clone; N., number; T-CLPD, chronic lymphoproliferative disorder of T cells; T-LGLL, T-cell large granular lymphocytic leukemia; TE, terminal effector.

In order to better define the utility of the TRBC1-FCM approach for high-sensitive assessment of T-cell clonality in T-LGLL in the clinical settings and improve its specificity for detection of (small) LGL clones, we subsequently combined the TRBC1 assay with the analysis of the TCRVβ repertoire and/or the presence of aberrant phenotypes by FCM. For this purpose, a second series of PB samples from 12 individuals (six HD, two TαβCD8-HDc and four TαβCD8-LGLL) was studied. Importantly, the simultaneous staining for T-cell maturation-associated markers and the TCRVβ repertoire allowed for the first time the detailed analysis of the TRBC1 expression profile together with the distribution of the different TCRVβ families, among normal/reactive CD28^−^ EM and TE Tαβ cells. It should be noted that normal Tαβ cells and their major subpopulations of TαβCD4 and TαβCD8 cells are known to display a variable distribution for the different TCRVβ families by FCM, and the TCRVβ antibodies included in the kit used here cover approximately 70–75% of the whole TCRVβ repertoire [24]. As expected, the more mature CD28^−^ EM and TE compartments of blood T cells showed a more restricted TCRVβ repertoire than total Tαβ and their major TαβCD8 and TαβCD4 cell subsets (Figure S1). The narrower TCRVβ repertoire observed for the more mature compartments of blood Tαβ cells is most likely due to the accumulation of effector T cells with a more restricted TCR repertoire, which is specific to a relatively limited number of antigens that these cells have recently encountered at these stages of maturation, in line with previous observations reported on activated T cells [25]. Further subsetting of mature CD28^−^ EM and TE Tαβ cells expressing individual TCRVβ families into TRBC1^+^ and TRBC1^−^ cells showed significantly higher absolute counts of clonal TRBC1^+^ or TRBC1^−^ Tαβ and TαβCD8 cells in T-LGLL and HDc (32–5515 cells/μL) compared to those identified among polyclonal TRBC1^+^ and TRBC1^−^ total Tαβ (0–25 cells/μL) and TαβCD8 (0–21 cells/μL) cells from normal/reactive blood (Figure 2A,C). Therefore, the absolute number of cells identified by a combination of markers that more precisely defines the TRBC1 expression profile within Tαβ cells expressing a given TCRVβ family (within a specific maturation stage) provides higher sensitivity and specificity for the detection of clonal T cells and improves the accuracy of the classical TRBC1-FCM approach for the detection of clonal Tαβ-LGL.

Subsequent analysis of the percentage of TRBC1^+^ cells per TCRVβ family within polyclonal LGL (Figure 2B,D) revealed that a polytypic/bimodal TRBC1 expression pattern was preserved for each TCRVβ family among the total Tαβ cells (range: 38–55%) [13,14]. In contrast, highly variable and more extreme TRBC1^+^ percentages were observed when the analysis was restricted to the more mature (CD28^−^ EM and TE) compartments of total Tαβ and TαβCD8 normal/reactive cells expressing individual TCRVβ families (range: 0% to 100% for all TCRVβ families, except for TCRVβ5.3^+^ cells within the total Tαβ cells and the TCRVβ5.3^+^, TCRVβ7.2^+^, TCRVβ13.2^+^ and TCRVβ14^+^ cells among TαβCD8 cells). This translated into a substantial overlap, with the percentage of TRBC1^+^ cells observed for clonal Tαβ-LGL (either <1.6% or >97.7% of TRBC1^+^ cells; Figure 2B,D). However, it should be noted that clonal Tαβ-LGL from half (3/6) of the T-LGLL/HDc investigated showed an aberrant phenotype that would allow their unequivocal identification and discrimination from residual polyclonal T cells (Table S3). These results indicate that the identification of clonality among Tαβ-LGL based on the pattern of expression of TRBC1 among cells expressing a single TCRVβ family should be based on the combined assessment of the absolute cell counts and/or the expression of aberrant phenotypes.

## 4. Conclusions

Here, we confirm and expand on previous observations about the clinical utility of the TRBC1-FCM assay for the rapid and easy assessment of T-cell clonality in the blood of individuals presenting with LGL lymphocytosis who are suspicious of T-LGLL based on the demonstration of altered (increased or decreased) percentages of TRBC1^+^ Tαβ cells (around either 0 or 100%). In contrast, our data indicate that, in the absence of lymphocytosis (or if TαβCD4-LGLL is suspected), the detection of increased absolute cell counts of subpopulations of T-LGL expressing individual TCRVβ families is required for the detection of T-cell clonality by the TRBC1-FCM assay; therefore, a more precise definition of T-LGL populations by TCRVβ provides the bases for the highly sensitive detection of T-cell clonality by FCM, even in the absence of aberrant phenotypes. Further comparisons of the TRBC1 assay here proposed against conventional T-cell clonality molecular approaches in a larger series of T-LGLL/HDc vs. normal/reactive PB samples covering the whole TCRVβ repertoire are required to confirm our preliminary findings.

## Data Availability

The data presented in this study are available in this article (and Supplementary Materials).

## Supplementary Materials

The following are available online at https://www.mdpi.com/article/10.3390/cancers14020408/s1: Protocol S1: Combining sample aliquots stained with a CD45 antibody conjugated to 8 different fluorochromes into only two antibody combinations ready to be measured in the flow cytometer, Table S1: Panels of fluorochrome-conjugated antibody reagents used in this study, Table S2: Sources and specificities of the monoclonal antibody reagents used in this study, Table S3: Detailed immunophenotypic features of T-cell subsets showing extreme TRBC1^+^ percentages within the more mature polyclonal and monoclonal Tαβ-cell populations expressing a specific TCRVβ family, Figure S1: Distribution of T cells expressing different TCRVβ families among total Tαβ cells and their Tαβ CD8^+^ and Tαβ CD4^+^ cell subsets and their maturation-associated stages of CD28^−^ effector memory and terminal effector cells as identified in the blood of healthy donors (*n* = 6).
