# Supplementary material for: High-Sensitive TRBC1-Based Flow Cytometric Assessment of T-Cell Clonality in Tαβ-Large Granular Lymphocytic Leukemia
Source: Cancers (Basel). 2022 Jan 14;14(2):408. doi: 10.3390/cancers14020408 (PMC8773687; doi:10.3390/cancers14020408)
Supplement: Supplementary file 1 [file cancers-14-00408-s001.zip › cancers-1536210-supplementary.pdf]

cancers

MDPI

Supplementary Material: High-Sensitive TRBC1-Based Flow Cytometric Assessment of T-Cell Clonality in Tαβ-Large Granular Lymphocytic Leukemia

Noemí Muñoz-García, F. Javier Morán-Plata, Neus Villamor, Margarida Lima[Ŧ], Susana Barrena, Sheila Mateos, Carolina Caldas, Jacques J.M. van Dongen, Alberto Orfao and Julia Almeida

Protocol S1. Combining sample aliquots stained with a CD45 antibody conjugated to 8 different fluorochromes into only two antibody combinations ready to be measured in the flow cytometer.

1. Prepare 1 to 8 tubes with 100 μL of peripheral blood
2. Add the appropriate volume of each TCRVβ, CD45, and TRBC1 antibodies per tube (as described in Supplementary Table 1, Panel II) in combination with 50 μL/tube of Brilliant Stain Buffer (Becton/Dickinson Biosciences (BD), San Jose, CA)
3. Mix well, preferably by gently vortexing
4. Incubate for 30 min at room temperature (RT) protected from light
5. Add 2 mL of washing buffer to the cell pellet
6. Mix well, preferably by gently vortexing
7. Centrifuge for 5 min at 540 g
8. Discard the supernatant using a Pasteur pipette or vacuum system without disturbing the cell pellet, leaving approximately 50 μL residual volume in each tube
9. Mix well, preferably by gently vortexing
10. Combine cells from tubes 1-4 and from tubes 5-8 into two single tubes, respectively; for this purpose, wash the 8 tubes from the first set of tubes with washing buffer to recover all cells that might have been left in the original tubes.
11. Centrifuge for 5 min at 540 g
12. Discard the supernatant using a Pasteur pipette or vacuum system without disturbing the cell pellet, leaving approximately 50 μL residual volume in each tube
13. Mix well, preferably by gently vortexing
14. Add the appropriate volume of the remaining antibodies to the two tubes
15. Mix well, preferably by gently vortexing
16. Incubate for 20 min at RT protected from light
17. Add 2 mL of 1X FACS Lysing Solution —10X FACS Lysing Solution (BD) diluted 1/10 vol/vol in distilled water, following the recommendations of the manufacturer—
18. Mix well, preferably by gently vortexing
19. Incubate for 15 min at RT protected from light
20. Centrifuge for 5 min at 540 g
21. Discard the supernatant using a Pasteur pipette or vacuum system without disturbing the cell pellet, leaving approximately 50 μL residual volume in each tube
22. Mix well, preferably by gently vortexing
23. Add 2 mL of washing buffer to the cell pellet
24. Mix well, preferably by gently vortexing
25. Centrifuge for 5 min at 540 g
26. Discard the supernatant using a Pasteur pipette or vacuum system without disturbing the cell pellet, leaving approximately 50 μL residual volume in each tube
27. Mix well, preferably by gently vortexing
28. Resuspend the cell pellet in 200 μL of acquisition buffer

29. Acquire the cells (preferably) immediately after staining or store at 4ºC for a maximum of 1 hour until measured in the flow cytometer

Table S1. Panels of fluorochrome-conjugated antibody reagents used in this study.

Panel I. Analysis of TRBC1 expression on different maturation-associated subsets of total Tαβ-cells and their major subsets

BUV661	BUV737	BV421	PacB	BV510	BV605	BV650	BV711	BV786	FITC	PE	PerCP Cy5.5	PECF594	PECy7	AF700	APC Vio770
CD7	TRBC1	CD27	CD2	CD45RA	CD4	CD62L	CD16	CD3	CD57	cyGra	CD28	CD8	TCRγδ	CD45	CD56

Panel II. Analysis of TRBC1 expression per TCRVβ-family (using the *IOTest® Beta Mark TCR Vβ Repertoire Kit-Beckman Coulter*) **among different maturation-associated subsets of Tαβ-cells**

BUV395	BUV661	BUV805	BV421	PacB	BV480	BV510	BV605	BV650	BV786	FITC	PE	PerCP	PerCP Cy5.5	PECy7	PE Vio615	Dy634	APC R700	APCH7
CD8	CD7	CD45	CD27	CD2		CD45RA	CD4	CD62L	CD3	TCRVβ A			CD28	TCRγδ	NKp80	TRBC1	CD5	
CD8	CD7		CD27	CD2	CD45	CD45RA	CD4	CD62L	CD3	TCRVβ B			CD28	TCRγδ	NKp80	TRBC1	CD5	
CD8	CD7		CD27	CD2		CD45RA	CD4	CD62L	CD3	TCRVβ C		CD45	CD28	TCRγδ	NKp80	TRBC1	CD5	
CD8	CD7		CD27	CD2		CD45RA	CD4	CD62L	CD3	TCRVβ D			CD28	TCRγδ	NKp80	TRBC1	CD5	CD45
CD8	CD7	CD45	CD27	CD2		CD45RA	CD4	CD62L	CD3	TCRVβ E			CD28	TCRγδ	NKp80	TRBC1	CD5	
CD8	CD7		CD27	CD2	CD45	CD45RA	CD4	CD62L	CD3	TCRVβ F			CD28	TCRγδ	NKp80	TRBC1	CD5	
CD8	CD7		CD27	CD2		CD45RA	CD4	CD62L	CD3	TCRVβ G		CD45	CD28	TCRγδ	NKp80	TRBC1	CD5	
CD8	CD7		CD27	CD2		CD45RA	CD4	CD62L	CD3	TCRVβ H			CD28	TCRγδ	NKp80	TRBC1	CD5	CD45

For all tubes, "stain & lyse" EuroFlow SOPs were used (www.EuroFlow.com), with the modifications described in Supplementary Protocol 1. Abbreviations (alphabetical order): APC, allophycocyanin; H7, Hilite®7; BD, Becton/Dickinson Biosciences; BV, Brilliant Violet™; cy, cytoplasmic; Dy, dyomics; FITC, fluorescein isothiocyanate; Gra, granzyme B; PacB, Pacific Blue™; PE, phycoerythrin; Cy5.5, cyanin 5.5; Cy7, cyanin 7; PerCP, peridinin-chlorophyl protein; TCR, T-cell receptor.

Table S2. Sources and specificities of the monoclonal antibody reagents used in this study.

Marker	Fluorochrome	Clone	Manufacturer	Volume (μL)
CD2	PacB	TS1/8	BioLegend	1
CD3	BV786	SK7	BD	1
CD4	BV605	SK3	BD	1
CD5	APCR700	UCHT2	BD	3
CD7	BUV661	M-T701	BD	0.5
CD8	BUV395	RPA-T8	BD	5
CD8	PECF594	RPA-T8	BD	1
CD16	BV711	3G8	BD	2.5
CD27	BV421	MT271	BD	2
CD28	PerCPCy5.5	CD28.2	BioLegend	5
CD45	BUV805	HI30	BD	5
CD45	BV480	HI30	BD	5
CD45	PerCP	HI30	BioLegend	5
CD45	AF700	HI30	BD	2.5
CD45	APCCy7	MEM-28	ExBio	5
CD45RA	BV510	HI100	BD	2.5
CD56	APCVio770	REA196	Miltenyi	2
CD57	FITC	HNK1	BD	10
CD62L	BV605	DREG56	BioLegend	2.5
CD62L	BV650	DREG56	BioLegend	2.5

Granzyme B	PE	GB11	Sanquin	5
NKp80	PEVio615	REA845	Miltenyi	2
TCRγδ	PECy7	11F2	BD	1
TRBC1	Dy634	JOVI-1	Immunostep	0.5
TRBC1	BUV737	JOVI-1	BD	1
TCRVβ 5.3	PE	3D11	Beckman Coulter	10 (Tube A)
TCRVβ 7.1	PE + FITC	ZOE	Beckman Coulter	
TCRVβ 3	FITC	CH92	Beckman Coulter	
TCRVβ 9	PE	FIN9	Beckman Coulter	10 (Tube B)
TCRVβ 17	PE + FITC	E17.5F3	Beckman Coulter	
TCRVβ 16	FITC	TAMAYA1.2	Beckman Coulter	
TCRVβ 18	PE	BA62.6	Beckman Coulter	10 (Tube C)
TCRVβ 5.1	PE + FITC	IMMU157	Beckman Coulter	
TCRVβ 20	FITC	ELL1.4	Beckman Coulter	
TCRVβ 13.1	PE	IMMU222	Beckman Coulter	10 (Tube D)
TCRVβ 13.6	PE + FITC	JU74.3	Beckman Coulter	
TCRVβ 8	FITC	56C5.2	Beckman Coulter	
TCRVβ 5.2	PE	36213	Beckman Coulter	10 (Tube E)
TCRVβ 2	PE + FITC	MPB2D5	Beckman Coulter	
TCRVβ 12	FITC	VER2.32	Beckman Coulter	
TCRVβ 23	PE	AF23	Beckman Coulter	10 (Tube F)
TCRVβ 1	PE + FITC	BL37.2	Beckman Coulter	
TCRVβ 21.3	FITC	IG125	Beckman Coulter	
TCRVβ 11	PE	C21	Beckman Coulter	10 (Tube G)
TCR-Vβ 22	PE + FITC	IMMU546	Beckman Coulter	
TCR-Vβ 14	FITC	CAS1.1.3	Beckman Coulter	
TCR-Vβ 13.2	PE	H132	Beckman Coulter	10 (Tube H)
TCR-Vβ 4	PE + FITC	WJF24	Beckman Coulter	
TCR-Vβ 7.2	FITC	ZIZOU4	Beckman Coulter	

Abbreviations (alphabetical order): APC, allophycocyanin; H7, Hilite®7; BD, Becton/Dickinson Biosciences; BV, Brilliant Violet™; Dy, dyomics; FITC, fluorescein isothiocyanate; PacB, Pacific Blue™; PE, phycoerythrin; Cy5.5, cyanin 5.5; Cy7, cyanin 7; PerCP, peridinin-chlorophyll; TCR, T-cell receptor.

Table S3. Detailed immunophenotypic features of T-cell subsets showing extreme TRBC1^{+} percentages within the more mature polyclonal and monoclonal Tαβ-cell populations expressing a specific TCRVβ family.

n. sample	Study group	Maturation Stage	TCRVβ family	*n.* cells / µL	%TRBC1^{+}	Phenotype	Aberrant phenotype
#1	HD	TE	Vβ13.1^{+}	11	7.1%	CD2^{lo} CD3^{+} CD4^{-} CD5^{lo} CD7^{+} CD8^{+} CD45RA^{+} CD62L^{-}	No
#2	HD	TE	Vβ7.2^{lo}	12	14%	CD2^{lo} CD3^{lo} CD4^{-} CD5^{lo} CD7^{++} CD8^{+} CD45RA^{+} CD62L^{+} NKp80^{+}	No
#3	HD	TE	Vβ17$^{+/++}$	13	18%	CD2$^{lo/+}$ CD3^{+} CD4^{-} CD5^{+} CD7^{+} CD8^{+} CD45RA^{+} CD62L^{het}	No
#1	HD	TE	Vβ3$^{lo/+}$	14	91%	CD2^{+} CD3^{+} CD4^{+} CD5^{+} CD7^{-} CD8^{-} CD45RA^{lo} CD62L^{+}	No
#4	HD	TE	Vβ5.1^{++}	14	0.24%	CD2$^{lo/+}$ CD3^{+} CD4^{-} CD5^{+} CD7^{lo} CD8$^{-/lo}$ CD45RA^{+} CD62L^{+} NKp80^{+}	No
#2	HD	TE	Vβ22^{++}	15	0%	CD2$^{lo/+}$ CD3^{+} CD4^{-} CD5$^{-/lo}$ CD7^{lo} CD8^{+} CD45RA^{lo} CD62L^{-} NKp80^{het}	No
#3	HD	TE	Vβ3^{+}	16	2.2%	CD2^{lo} CD3^{+} CD4^{-} CD5^{+} CD7^{+} CD8^{+} CD45RA^{+} CD62L^{-}	No
#1	HD	TE	Vβ17^{+}	25	1.4%	CD2^{lo} CD3$^{+/++}$ CD4^{-} CD5^{lo} CD7^{+} CD8^{-} CD45RA^{++} CD62L^{-}	No
#1	HD	TE	Vβ14^{+}	26	93%	CD2^{+} CD3^{lo} CD4^{-} CD5^{-} CD7^{++} CD8^{lo} CD45RA^{lo} CD62L^{-}	No
#5	Reactive*	TE	Vβ13.1^{+}	19	99%	CD2^{+} CD3^{lo} CD4^{+} CD5^{++} CD7^{-} CD8^{-} CD45RA^{lo} CD62L^{het}	No
#5	HDc	TE	Vβ8^{lo}	33	98%	CD2^{lo} CD3^{lo} CD4^{-} CD5$^{-/lo}$ CD7^{lo} CD8^{+} CD45RA^{++} CD62L^{-}	Yes
#6	HDc	TE	Vβ22^{+}	184	99.9%	CD2^{+} CD3^{++} CD4^{-} CD5$^{-/lo}$ CD7$^{-/lo}$ CD8^{lo}	Yes

						CD45RA$^{+/++}$ CD57^{+} CD94^{+} cyGra^{+} cyPerf^{+}	
#7	LGLL	TE	Vβ22^{+}	350	1.6%	CD2^{+} CD3^{+} CD4^{-} CD5^{het} CD7^{lo} CD8^{+} CD45RA^{lo} CD57^{+} CD94^{-} cyGra^{+} cyPerf^{+}	No
#8	LGLL	TE	Vβ1^{+}	691	0.14%	CD2^{lo} CD3^{++} CD4^{-} CD5^{lo} CD7^{+} CD8^{+} CD45RA^{+} CD57^{+} CD94^{+} cyGra^{+} cyPerf^{+}	No
#9	LGLL	TE	Vβ16^{+}	929	98%	CD2^{lo} CD3^{+} CD4^{-} CD5^{lo} CD7^{-} CD8$^{-/lo}$ CD45RA^{+} CD57^{+} CD94^{-}	Yes
#10	LGLL	TE	Vβ14^{+}	5,516	99.6%	CD2^{+} CD3^{+} CD4^{-} CD5$^{-/lo}$ CD7$^{-/lo}$ CD8^{+} CD57^{het} CD94^{+} cyGra^{+} cyPerf^{+}	No

* Residual (reactive) polyclonal T$\alpha\beta$-cell populations from a HDc. HD were selected based on the absolute number of TE T$\alpha\beta$ cells (>10 cells/μL). Abbreviations (alphabetical order): cy, cytoplasmic; Gra, granzyme B; HD, healthy donor; HDc, healthy donor with a small T$\alpha\beta$-cell clone in blood; het, heterogeneous expression; lo, low expression; n., number; LGLL, large granular lymphocyte leukemia; Perf, perforin; TE, terminal effector.

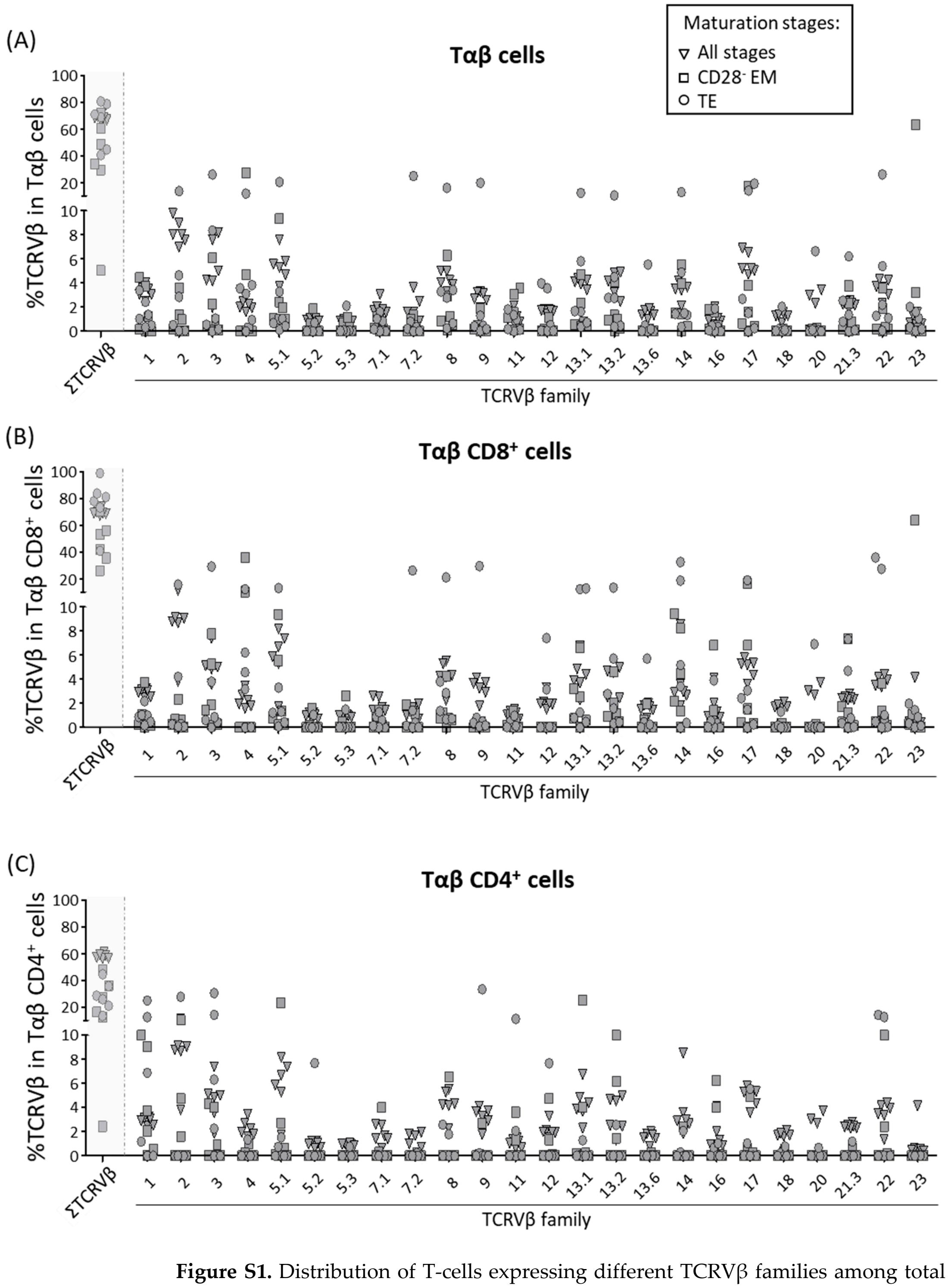

Figure S1. Distribution of T-cells expressing different TCRVβ families among total Tαβ cells and their Tαβ CD8+ and Tαβ CD4+ cell subsets and their maturation-associated stages of CD28- effector memory and terminal effector cells as identified in blood of healthy donors (n = 6). Abbreviations (alphabetical order): EM, effector memory; TE, terminal effector.
